# The Role of Follow-up Evaluation in the Diagnostic Algorithm of Idiopathic Interstitial Pneumonia: A Retrospective Study

**DOI:** 10.1038/s41598-019-42813-7

**Published:** 2019-04-23

**Authors:** Qian Han, Hong-yu Wang, Xiao-xian Zhang, Lu-lu Wu, Lu-lin Wang, Ying Jiang, Kui-miao Deng, Meng-meng Mao, Rong-chang Chen, Martin Kolb, Qun Luo

**Affiliations:** 1 Department of Medicine, The First Affiliated Hospital of Guangzhou Medical University, Guangzhou Institute of Respiratory Health, State Key Laboratory of Respiratory Disease, Guangzhou, China; 2 Department of Medicine, Pathology and Molecular Medicine, McMaster University, Firestone Institute for Respiratory Health, Hamilton, Ontario Canada

**Keywords:** Respiratory tract diseases, Diagnosis

## Abstract

We aimed to evaluate the alteration of diagnosis of individual expert and multidisciplinary discussion (MDD) team in the longitudinal diagnostic assessment of idiopathic interstitial pneumonia (IIP). The retrospective analysis included 56 patients diagnosed as IIP by The First Affiliated Hospital of Guangzhou Medical University with follow-up visits during Jan 1^st^ to Aug 31^st^ 2014. Each expert was provided information in a sequential manner and was asked to assign an individual diagnosis and an MDD diagnosis after group discussion. The level of agreement among individual experts and between different visits was calculated by kappa and the agreement between individual specialist and MDD team with different consensus levels was measured by weighted-kappa coefficients. Follow-up data changed the original clinical diagnosis and MDD diagnosis in 24.1% and 10.7% of all cases, respectively, and clinician and MDD consensus level in 55.4% and 25.0%, respectively. The diagnostic performance of individual clinicians or radiologist was closer to that of the MDD compared with the pathologist, and follow-up further increased the agreement. The longitudinal evaluation of patients with IIP improved the inter-observer agreement in a multidisciplinary team. The performance of individual clinicians or radiologist was approaching the accuracy of multidisciplinary team when provided with follow-up data.

## Introduction

Interstitial lung diseases (ILDs) represent a diverse group of lung diseases with varied etiology, pathological change, treatment and prognosis. The establishment of an accurate diagnosis is essential to determine therapeutic interventions and prognosis for a given patient. The multidisciplinary diagnostic approach has been advocated since 2002 by the joint statement of the American Thoracic Society (ATS) and the European Respiratory Society (ERS), which includes the interaction between expert clinicians, radiologists and pathologists^1^. The cooperative diagnostic mode was re-emphasized in the update on idiopathic pulmonary fibrosis (IPF) guideline in 2011^2^ and on the classification of idiopathic interstitial pneumonia (IIP) in 2013^3^. However, the composition of multidisciplinary discussion (MDD) team and the implementation of the discussion still remains controversial^4^. Most clinicians in daily practice are tasked with evaluating ILD patients without access to qualified radiological and pathological expertise. On the other hand, MDD does not always generate a confident diagnosis. A provisional or a working diagnosis, drawn on that moment, may change over time. Previously unrecognized exposure, serological alteration, response to treatment and disease behavior per se may change the diagnosis and thus patient management. Several studies have evaluated the inter-observer and inter-multidisciplinary team agreement in the diagnosis of ILDs^5–8^, but sparse evidence existed assessing the role of longitudinal follow-up in the diagnostic pathway, i.e. the intra-observer and intra-MDD team agreement between the first and follow-up presentations as well as the alteration of inter-observer agreement over time. In the current study, we aimed to evaluate the performance of each expert participant in the MDD team and the team as a whole in the longitudinal diagnosis process, thereby providing some evidence on the role of follow-up in the diagnostic algorithm of IIP.

## Results

A total of 140 consecutive patients were admitted and identified as IIP between January 1st, 2014 and Aug 31st, 2014, from which 56 (40.0%) had previous referral(s) and included in this study. There were 24 (42.9%) males and 32 (57.1%) females aged 56.9 ± 12.6 yrs. Twenty-one (37.5%) patients underwent SLB and 20 (35.7%) patients TBLB. No biopsy was done in 15 (26.8%) patients. All initial diagnoses were made by the MDD in the host institute. The mean follow-up duration was 7 months (1–36 months), during which 51 (91.1%) patients received steroid treatment (Predisolone ≤0.5 mg/kg).

The first-choice diagnoses given on 56 patients by 4 clinicians, 1 radiologist and the multidisciplinary team on the first and follow-up admissions are shown in Table 1 (The diagnoses given by 4 clinicians on 56 cases are combined together into 224 diagnoses). The most common diagnosis made by MDD on both visits was non-specific interstitial pneumonia (NSIP), followed by cryptogenic organizing pneumonia (COP), respiratory bronchiolitis-associated interstitial lung disease (RB-ILD)/desquamative interstitial pneumonitis (DIP) and IPF. Definite pathological diagnoses could only be made in 18 of 56 (32.1%) patients, which were totally from SLB but not TBLB samples (data not shown). The distribution was more similar between MDD team and clinicians compared to the radiologist and pathologist.

**Table 1:** Diagnoses given by clinicians, radiologist, pathologist and multidisciplinary team on each admission.

Initial diagnosis							Follow-up diagnosis			
	Clinicians*		Radiologist		Pathologist	Final dx		Clinicians*	Radiologist	Final dx
	Total (n = 224)	With Pathology (n = 72)	Total (n = 56)	With pathology (n = 18)	Total (n = 18)	Total (n = 56)	With pathology (n = 18)	Total (n = 224)	Total (n = 56)	Total (n = 56)
IPF	8.8	7.8	8.9	NA	16.7	7.1	5.6	5.9	5.4	7.1
NSIP	37.7	42.2	60.7	66.7	38.9	46.4	50	42.2	53.6	44.6
RB-ILD/DIP	7.4	20.3	8.9	16.7	11.1	9	22.3	6.9	5.4	7.2
COP	16.2	12.5	8.9	11.1	5.6	16.1	5.6	16.2	14.3	16.1
LIP	1	NA	1.8	NA	NA	3.6	NA	2.9	3.6	3.6
U-IIP	5.9	1.6	8.9	NA	NA	5.4	NA	5.4	8.9	5.4
HP	10.8	7.8	1.8	5.6	5.6	3.6	NA	8.3	7.1	5.4
CTD-ILD	10.8	7.8	0	0	16.7	5.4	5.6	11.3	0	8.9
Other ILD	1.5	NA	0	0	5.6	1.8	5.6	1	1.8	1.8

IPF: idiopathic pulmonary fibrosis; NSIP; non-specific interstitial pneumonia; RB-ILD/DIP: respiratory bronchitis interstitial lung disease/desquamative interstitial pneumonia; COP: cryptogenic organizing pneumonia; LIP: lymphocytic interstitial pneumonia; U-IIP: unclassifiable idiopathic interstitial pneumonia; HP: hypersensitivity pneumonitis; CTD-ILD: connective tissue disease related interstitial lung disease. *244 diagnoses in total given by clinicians on 56 cases.

**Figure 1:**
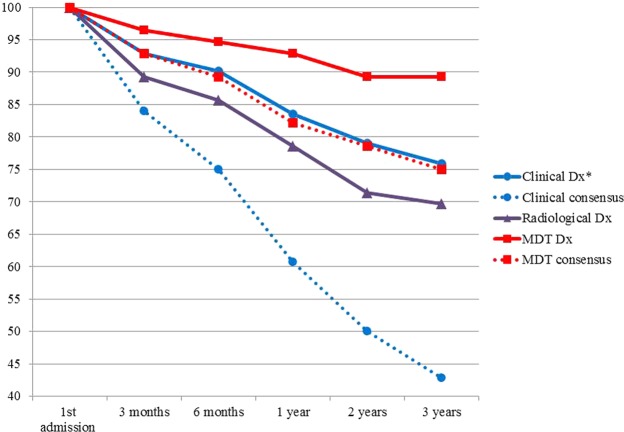
The alteration of diagnosis and consensus level with different intervals during follow-up. Dx: diagnosis; MDD: multidisciplinary discussion. *244 diagnoses in total given by clinicians on 56 cases. Follow-up data changed the original MDD diagnosis in 10.7% (6 of 56) patients and altered the final diagnostic consensus level in 25.0% of all cases (14 of 56); Follow-up data changed the original clinical and radiological diagnosis in 24.1% (54 of 224) and 30.4% (17 of 56) cases, respectively, and altered the clinician consensus level in 55.4% of all cases (31 of 56).

Follow-up data changed the original MDD diagnosis in 10.7% (6 of 56) patients and the original clinical diagnosis in 24.1% (54 of 224) cases (Fig. 1). The most commonly altered diagnosis among clinicians was IPF (9/18, 50.0%), followed by CTD-ILD (9/23, 39.1%), hypersensitivity pneumonitis (HP, 7/22, 31.8%) and COP (9/33, 27.3%). The change of consensus levels among clinicians and among all observers in multidisciplinary team during the first admission and follow-up were demonstrated in Fig. 1 and Table 2. Follow-up data altered the final diagnostic consensus level in 25.0% of all cases (14 of 56), upgrading (from cannot agree to majority vote or full consensus, or from majority vote to full consensus, respectively) in 9 and downgrading (on the contrary to upgrading) in 5 cases, respectively. It induced a greater change for the inter-clinician consensus level (31 of 56, 55.4% of all cases), upgrading in 28 and downgrading in 3 cases, respectively.

**Table 2:** Inter-observer agreement of major individual diagnoses of interstitial lung diseases on each admission.

	1st admission			Follow-up	
	Clinicians	Clinicians -radiologist	All observers (n = 18)	Clinicians	Clinicians -radiologist
IPF	0.125	0.252	0.228	0.614	0.608
NSIP	0.382	0.416	0.347	0.633	0.616
RB-ILD/DIP	0.760	0.689	0.610	0.921	0.876
COP	0.606	0.621	0.519	0.896	0.906
HP	0.192	0.167	0.251	0.440	0.482
CTD-NSIP	0.237	0.160	0.101	0.427	0.287
Total	0.349	0.369	0.323	0.620	0.615

IPF: idiopathic pulmonary fibrosis; NSIP; non-specific interstitial pneumonia; RB-ILD/DIP: respiratory bronchitis interstitial lung disease/desquamative interstitial pneumonia; COP: cryptogenic organizing pneumonia; HP: hypersensitivity pneumonitis; CTD-NSIP: connective tissue disease related non-specific interstitial pneumonia.

The inter-observer agreement between clinicians, radiologist and pathologist for first-choice diagnoses on both visits was demonstrated in Table 3. The overall inter-observer agreement for initial diagnoses was fair (κ ranging from 0.323 to 0.369). The inter-observer agreement was substantial for RB-ILD/DIP, moderate to substantial for COP, fair for NSIP and slight to fair for HP and IPF. The overall or subtype inter-observer agreement for follow-up diagnoses was increased not only between clinicians but also between clinicians and radiologist, with perfect agreement for RB-ILD/DIP and COP and the most striking increase for IPF.

**Table 3:** Agreement of individual clinicians, radiologist and pathologist with final diagnosis on each admission.

	1^st^ admission (κw)	Follow-up(κw)
Clinician A vs final	0.550 (0.375–0.726)	0.901 (0.801–1.001)
Clinician B vs final	0.606 (0.435–0.776)	0.842 (0.719–0.965)
Clinician C vs final	0.572 (0.358–0.785)	0.731 (0.536–0.925)
Clinician D vs final	0.400 (0.226–0.573)	0.728 (0.579–0.876)
Radiologist vs final	0.549 (0.367–0.731)	0.740 (0.586–0.895)
Pathologist vs final (n = 18)	0.295 (−0.035–0.624)	0.264 (−0.06–0.588)

The agreement of initial diagnoses between MDD and individual specialist was moderate for the clinician and the radiologist (κ ranging from 0.400 to 0.606), and the follow-up further improved the agreement to substantial to perfect (κ ranging from 0.728 to 0.901) (Table 4). The intra-observer agreement was moderate to substantial for all individual observers (κ ranging from 0.528 to 0.747) and perfect for MDD (κ = 0.857) (Table 4). However, the pathological diagnoses only showed fair agreement with either initial or follow-up final diagnoses (κ = 0.295 and 0.264, respectively) (Table 4).

**Table 4:** Intra-observer agreement for clinicians, radiologist and multidisciplinary team on each admission.

	Total (κ) (n = 56)	Without pathology (κ) (n = 38)	With pathology (κ) (n = 18)
Clinician A	0.632 (0.493–0.771)	0.612 (0.426–0.798)	0.651 (0.332–0.846)
Clinician B	0.747 (0.614–0.880)	0.765 (0.612–0.918)	0.695 (0.438–0.952)
Clinician C	0.714 (0.528–0.900)	0.574 (0.325–0.823)	1
Clinician D	0.573 (0.424–0.722)	0.528 (0.348–0.708)	0.634 (0.365–0.903)
Radiologist	0.528 (0.359–0.697)	0.460 (0. 262–0.658)	0.679 (0.379–0.979)
Final diagnosis	0.857 (0.749–0.965)	0.891 (0.775–1.007)	0.706 (0.559–1.001)

## Discussion

We showed in our current study that the diagnosis of IIP is a dynamic process undergoing substantial change over consecutive follow-up at regular intervals. Access to follow-up data greatly increased agreement not only between clinicians but also between radiologists and clinicians. Follow-up data changed the original MDD diagnosis in 10.7% and the final diagnostic consensus level in 25.0% of all cases; it altered the original clinical diagnosis in 24.1% and the clinician consensus level in 55.4% of all cases. Compared to the pathologist, the diagnostic performance of individual clinicians or radiologist on IIPs was closer to that of the MDD and follow-up data further increased the agreement.

Although SLB is recommended in the diagnosis of undefined ILDs, it is not without risks and mortality and clinicians should weigh the diagnostic effectiveness and potential risks when making decisions^9–12^. The diagnostic accuracy of pathology depends not only on the pattern recognition but also on the biopsy location. It is known that it can be difficult to distinguish a UIP with NSIP pattern based on histology, and perhaps more difficult with knowledge of the lobar histopathological variability in UIP and NSIP in the same lung^7,13^. This finding was confirmed by studies showing that approximately half of cases with pathological diagnosis of UIP were classified into low-level certainty by expert radiologists and pulmonologists with HP, CTD-ILD and NSIP as the most common differential diagnoses^14^. Similarly, Burge *et al*. found that MDD changed the original histological diagnoses in 30% and strengthened the diagnoses from probable to confident in a further 17% of 71 cases^15^. In our study, the agreement was slight between the pathological result and MDD: In 3 cases pathologically diagnosed as usual interstitial pneumonia (UIP), two were confirmed as NSIP/CTD-NSIP with MDD; in 7 cases pathologically diagnosed as NSIP, one was confirmed as DIP and one as COP by MDD. Furthermore, the agreement between pathologist and MDD remained the same during follow-up and 1^st^ admission (κw = 0.264 *vs* 0.295), and the intra-multidisciplinary team agreement was even higher in cases without pathological results ((κ = 0.891 *vs* 0.706), indicating the individual experts may not stick to the original diagnoses during the longitudinal evaluation even with the pathological opinion. Together with published literature, our results demonstrated the limitation of pathological diagnosis, when separated from clinical data, in the diagnosis of ILD.

International guidelines emphasized the importance of a multidisciplinary diagnostic process^2^, while it has not been firmly validated and does not guarantee diagnostic accuracy. Firstly, the diagnostic process may be affected by individual experts with different levels of experience in the diagnosis of ILDs. A study comparing inter-observer agreement within as well as between community and academic locations found that there was overall poor agreement between academic and community centers, yet better final agreement between academic centers^6^. All participants in the present study are from tertiary hospitals in China specialized in the diagnosis and management of ILDs for at least 10 years, therefore minimizing the observer bias to a certain extent. Secondly, a definite diagnosis cannot always be made even with MDD, the working diagnosis may be achieved to guide the management of the patients and should be re-reviewed in the longitudinal course^4,12,16^.

In our study, follow-up data changed the original clinician diagnosis in 54 out of 224 cases, and the diagnoses more prone to be affected by follow-up data were IPF (50.0%) and CTD-ILD (39.1%). The change of diagnoses, although difficult to attribute, may mainly be derived from the disease behavior with or without treatment. The differential diagnosis between IIPs with those related to CTDs is not always straightforward when extrapulmonary signs and serological abnormalities are subtle, while these may turn obvious with the time being^17,18^.

Our study showed that the inter-observer agreement was moderate to substantial for RB-ILD/DIP and COP. The relatively high agreement on RB-ILD/DIP is likely due to the fact that a history of smoking combined with typical radiological changes, i.e. bronchial thickening followed by centrilobular nodules and ground-glass opacity (GGO) in RB-ILD and extensive bilateral GGO with a peripheral and basal predominance in DIP, is usually sufficient to diagnose these ILD subtypes causally associated with smoking^19^. Moreover, the improvement observed on the follow-up admission following smoking cessation and corticosteroid treatment in most cases would allow physicians more confident on these diagnoses.

On the other hand, it was surprising that, distinct with other studies^5,8^, the κ scores for IPF failed to attain a satisfactory level even with evidence-based diagnostic criteria. We combined IPF diagnoses with different confidence levels (definite, probable or possible) due to the relatively low prevalence of first-choice diagnoses, and approximately half (8 in 18) cases were assigned as probable or possible IPF by one clinician while diagnosed as NSIP by other clinicians as well as by MDD. When provided with follow-up data, the most striking increase in κ scores among observers was found for IPF, and this increase was mainly derived from the change of clinical diagnoses to NSIP from those 8 cases of low-confident IPF. The boundary between low-confident IPF and NSIP is ambiguous, and the decision is mostly driven by clinical reasoning and intention of clinicians on the treatment algorithm^20^. Since this study was performed just before approval of anti-fibrotic drugs in China, the acceptance of these drug was limited, and making a diagnosis of IPF would just have denied patients the opportunity to receive steroid treatment. In these situations, clinicians might have preferred to diagnose NSIP rather than IPF thereby offering patients the opportunity of treatment with steroid/immunosuppressant. Additionally, the alteration of low-confidence IPF diagnoses to NSIP during follow-up indicated that dynamic monitoring of patients adds further diagnostic value in the subgroup of patients with significant uncertainty.

Although follow-up data only altered MDD diagnoses in 6 of 56 cases (10.7%), it changed the consensus level in 14 cases (25.0%). Furthermore, when considering clinician experts as a group, the alteration of consensus level was more remarkable (31 of 56 cases, 55.4%), indicating that longitudinal evaluation could increase individual diagnostic confidence albeit without effect on final decision. It is of great importance in clinical practice because some physicians are required to evaluate patients suspected of ILDs with restricted access to appropriate radiological and pathological expertise. While studies on diagnostic discrepancies between ILD experts in isolation and their respective MDD showed contradictory results^8,16^, our study demonstrated additional value of follow-up data. We showed moderate agreement between clinicians and the MDD (κ = 0.400–0.606) during the initial referral, which may be, at least partly, due to the composition of MDD and the implementation of discussion procedure different from typical setting. The inclusion of more clinicians (4 from different institutions) than counterparts (one radiologist and one pathologist) was because we mainly focused on the longitudinal evaluation during which, in the real-life setting, only clinicians have a face-to-face consultation with patients together with clinical and radiological data. The increase of agreement from moderate to substantial (κ = 0.728–0.901) between clinicians and MDD further supported the role of follow-up to refine the diagnostic process even with clinicians working in isolation.

Our paper has several limitations. Since we performed our study, an ATS/ERS task force released the diagnostic criteria for patients with IIP and an autoimmune “flavour” as interstitial pneumonia with autoimmune features (IPAF)^21^. Our study was performed in the “pre-IPAF” era and some cases initially diagnosed as i-NSIP/COP or CTD-NSIP/-OP may be diagnosed as IPAF according to the present classification criteria. However, the follow-up of patients belonging to this subtype would allow physicians to obtain the knowledge on the natural progression and response to treatment. Second, this study was performed in the host institute for 5 days including 4 pulmonologists from different ILD tertiary referral centers, which may partly explain the relatively small sample size. However, the composition of MDD team, emphasizing more on clinicians, serves to evaluate the follow-up in the diagnostic algorithm of ILDs. Third, the retrospective nature of our study precluded us to demonstrate the mechanism for the improvement of inter-observer agreement. The well-designed prospective study with uniform treatment algorithm and regular follow-up interval will contribute to this. Lastly, this study merely involved patients from a single-center and specialists restricted in China. However, as discussed, the diagnosis and management of patients with ILDs may be affected by medical insurance coverage and population recognition, which, although of great importance, is difficult to assess. Our study still sheds new light on the diagnostic algorithm of ILD in Chinese population.

In conclusion, our data showed that the dynamic evaluation of patients with IIP improved the inter-observer agreement in a MDD. We showed low levels of the initial inter-observer agreement for IPF. This might be related to availability and insurance coverage of novel antifibrotic therapies. Moreover, the performance of individual clinicians was approaching the accuracy levels of multidisciplinary teams when they were provided with follow-up data. The pathological conclusion, albeit straightforward, should always be interpreted in the context of the opinions from the clinicians and radiologist. Future prospective studies involving multi-centers are needed to explore the appropriate composition of multidisciplinary team and follow-up intervals in patients with ILD in China.

## Methods

### Patient selection

This is a single center, retrospective, observational case-cohort study, for which we selected consecutive patients who presented to the ILD center of the First Affiliated Hospital of Guangzhou Medical University between January 1st, 2014 and Aug 31st, 2014 (Guangzhou, China, host institution), each with final diagnosis as IIP by specialist expertise. The patients admitted for follow-up during that period were included in the study and the first admission data was extracted as well. All patients had comprehensive diagnostic information (clinical data, HRCT, spirometry test and, if available, lung biopsy) provided by the host institution. The experimental protocol was approved by Ethics Boards of the First Affiliated Hospital of Guangzhou Medical University. All methods were carried out in accordance with institutional guidelines and regulations. All subjects signed written informed consent in the current study.

### Study protocol

Both the individual diagnosis and multidisciplinary discussion was held in the host institution during Jan 18th to Jan 22nd, 2015. Four respirologists (LQ Lfrom the host institution, DHP from China-Japan Friendship Hospital, XZJ from Peking Union Medical College Hospital and LHP from Shanghai Pulmonary Hospital), one respiratory radiologist (Q.S Zeng from the host institution) and one thoracic pathologist (Y.Y Gu from the host institution) from 4 different tertiary hospitals in China were invited and agreed to participate in the study, each was experienced in the diagnosis and management of ILDs for at least 10 years.

The clinical data included general information (age, sex, smoking history), exposure history (environmental, occupational, drug), history of established connective tissue disease (CTD), symptoms, signs and serological results implying CTD and spirometry data and was entered into case report forms (CRFs). The CRFs were designed and modified mainly based on American College of Chest Physicians (ACCP) ILD questionnaire (with CHEST copyright permission) that combined with an ILD questionnaires applied in The Firestone Institute for Respiratory Health (kindly provided by MK). This provides the integrity and consistency of the clinical data allowing for subsequent evaluation.

HRCT of the thorax was performed in the supine position during breath-holding at full inspiration, with 1 or 1.5-mm thick sections at 1-cm intervals throughout the entire thorax. No intravenous contrast was administered except when pulmonary embolism was highly suspected. Pathologists had access to all pathology data that were available in the form of digitalised slides, which were captured using Hamamatsu NanoZoomer HT (Hamamatsu Photonics KK) digital scanner and transformed into the tiff format.

**Figure 2:**
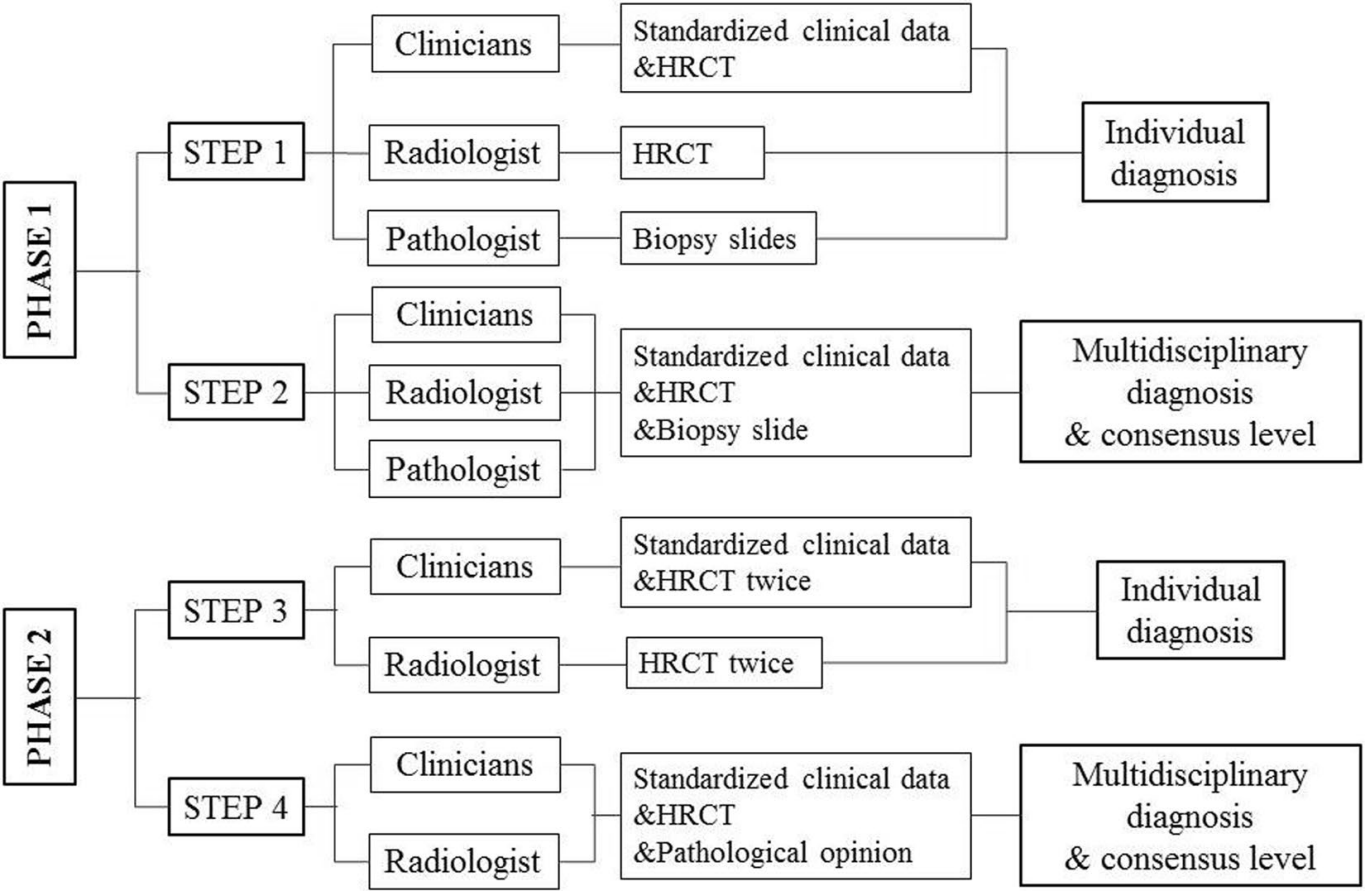
Schematic representation of the information presented to individual experts with different output at each step. Standardized clinical data: general condition (age, sex, smoking history), exposure history (environmental, occupational, drug), history of established connective tissue disease, symptoms, signs and serological results implying connective tissue disease and spirometry data input to the standardized form. HRCT: high-resolution computed tomography. The information presented in phase 2 includes both the first and follow-up visits.

The evaluation of each patient was divided into 2 phases and 4 steps (Fig. 2): At first, clinicians, radiologist and pathologist were asked to independently review the cases and assign the preliminary diagnoses for a given case. Clinicians were provided with comprehensive clinical data and HRCT images; radiologist and pathologist were only provided with general condition and the HRCT images (radiologist) or digitalized lung biopsy slides (pathologist, from transbronchoscopic lung biopsy, TBLB or surgical lung biopsy, SLB, if available).

For each patient, expert participants had to select up to three differential diagnoses on the diagnosis form. Terminology and diagnostic classification followed the update on the diagnosis of IPF in 2011 and the classification of IIP in 2013. In step 2, once individual diagnoses had been independently rendered, all clinicians, radiologist and pathologist assembled in an MDD team to review each case and assign a final consensus diagnosis. Clinical data, HRCT and digitalized lung biopsy slides were presented to all observers. Discussion started with displaying the individual preliminary diagnoses from panel members. The definite final diagnosis was attained when experts with different diagnostic impressions reached full consensus or majority vote and was recorded with different consensus extent. Moreover, the clinician consensus levels for each case were also recorded as full consensus (4:0), majority vote (3:1 or 2:1:1) or cannot agree (1:1:1:1).

Only clinicians and the radiologist were asked to participate in the second phase, during which they had to remain/alter their primary diagnoses to one secondary diagnosis. All observers were informed with the interval between the first and follow-up presentations, HRCT twice and their original diagnoses (including pathological opinions from the last phase); clinicians were additionally provided with the clinical information including change of symptoms, signs, serological and spirometry results as well as the treatment course inter-between. During the final step, the MDD including 4 clinicians and 1 radiologist was held to form a final diagnosis for each case with different consensus levels.

### Statistical analysis

We used kappa coefficients (κ) to determine the inter-observer agreement among individual specialists in diverse IIP subtypes and intra-observer agreement between visits. The alterations of diagnoses and consensus levels were plotted against follow-up intervals (3months, 6 months, 1st year, 2nd year and 3rd year) that are generally applied in our routine clinical practice. We used weighted-kappa coefficients (κw) to test the diagnostic agreement between individual specialist and multidisciplinary team with different consensus levels, i.e. full consensus or majority vote. Kappa coefficients were rated as almost perfect agreement (above 0.8), substantial agreement (scores between 0.6 and 0.8), moderate agreement (scores between0.4 and 0.6), fair agreement (scores between 0.2 and 0.4), slight agreement (scores between 0.0 and 0.2), and poor agreement (scores below 0.0). Statistical analyses were performed using Stata 14 (Stata Press, College Station, Texas, USA) and IBM SPSS Statistics 20.0 (IBM Corporation, Armonk, NY, USA).
